# Grain security assessment in Bangladesh based on supply-demand balance analysis

**DOI:** 10.1371/journal.pone.0252187

**Published:** 2021-05-26

**Authors:** Luguang Jiang, Si Wu, Ye Liu, Cheng Yang

**Affiliations:** 1 Institute of Geographic Sciences and Natural Resources Research, Chinese Academy of Sciences, Beijing, China; 2 College of Resources and Environment, University of Chinese Academy of Sciences, Beijing, China; University of Bern, SWITZERLAND

## Abstract

Ensuring the grain supply-demand balance and achieving grain security had been the main tasks for the government of Bangladesh. On the supply side, Bangladesh’s supply of grain products has increased substantially, with an average annual growth rate of 1.99 million tons in 1998–2018. Domestic grain production, especially rice production, accounted for the largest proportion in its structure. However, under the constraints of resources and environment, imports and international aid were needed to ensure a stable and sustainable grain supply. On the demand side, Bangladesh’s demand for grain products continued to grow at an average annual rate of 2.09 million tons and its structure was constantly diversified. In recent years, domestic grain production has fully met the grain demand for food use, but the overall grain supply dependence on foreign gradually increased. From the analysis of the influencing factors, the grain supply, especially the domestic production of rice and maize, had the greatest impact on the balance of grain supply-demand in Bangladesh. Moreover, multiple cropping index, chemical fertilizer application per hectare and irrigation rate were the three main factors affecting grain production. As a typical agricultural country, Bangladesh’s grain security was faced with challenges, such as high population density, insufficient cultivated land resources, international grain trade and frequent natural disasters. It is suggested that its government should strengthen scientific and technological research, adjust agricultural structure, improve the efficient utilization of agricultural resources and grain circulation systems, and balance the grain demand between food use and indirect use, so as to achieve complete grain self-sufficiency and overall grain security.

## Introduction

As a basic necessity, grain is the most important material basis to ensure the sustainable development of human beings and plays an irreplaceable fundamental role in supporting social and economic development [1]. Grain is one of the components of food, so grain security and food security have the same connotation [2]. Food security was first put forward in the International Agreement on World Food Security adopted by the Council of the Food and Agriculture Organization of the United Nations in 1974, which initially only meant that it could guarantee the basic survival and life of human beings. On 13 November 1996, the World Food Summit issued the Rome Declaration on World Food Security, which provided a comprehensive formulation of the concept of food security. Food security meant access by all people at all times to enough food for an active, healthy life [3, 4]. Nutritional security meant that in the daily life of human beings, there should be enough, balanced, and contain the nutritional elements necessary for human development, so as to achieve perfect food security. Grain security referred to the status in which the grain supply was evenly distributed in space and time to meet the basic needs of all people, and all people had easy access to grain that met the hygienic standards of nutrition and health [5]. The supplement and perfection of those concept reflected that people not only required grain to be sufficient in quantity and quality to maintain the basic survival of human needs, but also put forward higher standards and requirements for the availability, access, utilization and stability of grain security [6]. Therefore, national grain security was the most basic and important concept, which was the premise of comprehensive food security and personal nutrition security. With the continuous expansion of global cities and the growing nutritional needs of the global population and the spread of COVID-19 worldwide and the globe’s population lockdown, the global grain pattern presented a complex and changeable situation [7, 8]. And the issue of grain supply-demand has been the focus of attention all over the world [9, 10].

**Fig 1:**
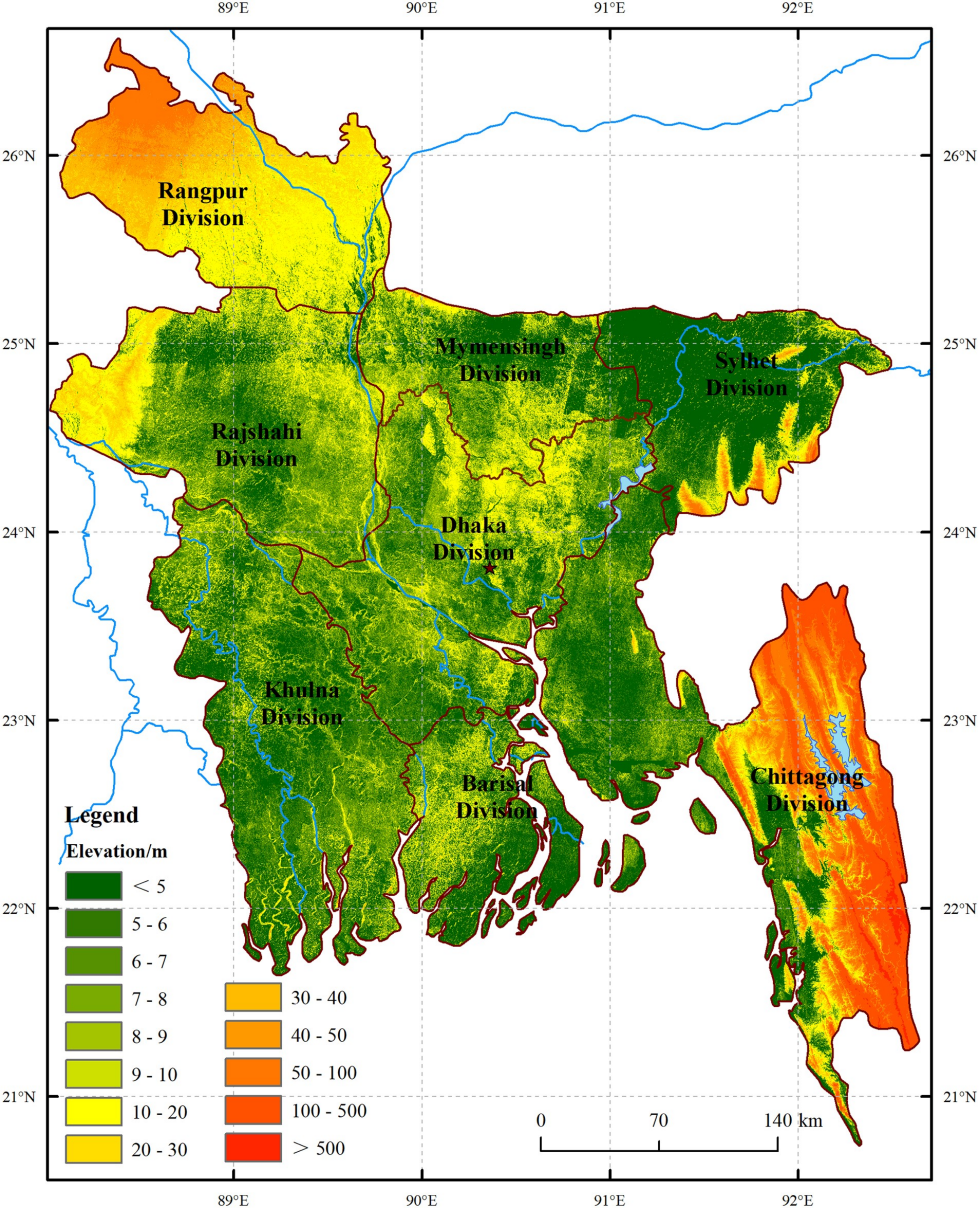
Location and digital elevation model of Bangladesh. The vector and elevation data used in this figure were obtained from Natural Earth (http://www.naturalearthdata.com) and Shuttle Radar Topography Mission (SRTM) dataset of United States Geological Survey (USGS) (http://eros.usgs.gov/). The figure was similar but not identical to the original image and used for the description of elevation and position of Bangladesh.

Bangladesh (Fig 1) was a country listed by Food and Agriculture Organization of the United Nations (FAO) as requiring external assistance to cope with severe localized grain insecurity caused by economic constraints, monsoon floods and high prices of the main staple grain [11]. Ensuring a balance between grain supply-demand had been a major priority since its independence in 1972 [12]. In the early days of the founding of Bangladesh, the slow economic development and rapid population growth made it impossible to balance the grain production capacity and demand. During that period, Bangladesh was a typical region of the world with high population density and grain insecurity. Around the 21th century, its political situation gradually stabilized, and the economic level and agricultural production capacity were greatly improved. Bangladesh Water Development Board (BWDB) began to promote the mechanization of irrigation in the main grain producing areas. The local governments introduced modern high-yielding new varieties of rice and potato, which greatly improved the agricultural cultivation methods and planting conditions. At the same time, Bangladesh had made considerable progress in controlling population growth. According to the Population and Housing Census and the United Nations Population Fund in 2011, its annual population growth rate was 1.37%, down from 1.58% in 2001. And the number of children per woman had reduced from 3.3 to 2.3, making Bangladesh a low fertility country [13]. In addition, Bangladesh had made considerable progress against human development indicators, but malnutrition resulting from poor dietary diversity and low micronutrient intakes remained entrenched [14]. Household nutrition insecurity remained a key public health problem in Bangladesh, with households suffering food shortages for an average of one quarter of the year [15]. In addition to grain, fish was central to the Bangladeshi diet and an irreplaceable animal-source food in the diet of millions [16]. With the dietary improvement, total grain demand would continue to increase in the coming decades [17]. However, more frequent and intense droughts and increasing temperatures, and the rising sea level might cause steady loss of the arable land, resulting in adverse impacts on grain supply [18, 19]. Under the influence of factors such as the continuous growth of population, the reduction of arable land, natural hazard and other factors, partial grain insecurity still existed in Bangladesh. As an important part of the Belt and Road Initiative and typical developing country, the situation of grain supply-demand and development potential of Bangladesh were of great significance to South Asia and even global grain security [20].

In recent years, many scholars have carried out a deep research on the impact of food production [21, 22] and food trade [23, 24] and the assessment and forecasting of food security [25] from a global and other large-scale perspective. However, in the study of Bangladesh, some scholars focused on the analysis of the prediction of cereals supply-demand [26], the influencing mechanism between dietary change and food supply [27], the impact of food shortage [28], and agricultural development model [29, 30]. At the same time, many scholars are concerned about nutritional insecurity in Bangladesh. They used small-scale survey data to focused on the analysis of the nutritional status of children and women and the relationship between them and food security [31, 32], dietary diversity and its influencing factors [33–35] and the impact of fishery development on nutrition security [15, 16].

Summarizing the previous research results, from the perspective of research content, previous researches mainly focused on food security and nutrition security, and seldom involved national macroscopic grain security, being short of research combining grain production, consumption and supply-demand. In terms of study area and research scale, the results of a large scale food situation assessment were less applicable to Bangladesh, and small-scale empirical studies of nutrition security had obvious limitations. The purpose of this paper was to explore the macroscopic grain security situation and its influencing factors in Bangladesh from the perspective of supply and demand, so as to enhance the pertinence, applicability and comprehensiveness of grain security research in Bangladesh. In the 1990s, Deng proposed the grey relational analysis as an impact assessment method, which measured the degree of similarity or difference between two sequences through the relational grade [36–38]. Based on its basic principles, it was a promising and effective method to assess the influencing factors of grain security. Based on the data of long time series (1998–2018), this paper innovatively used the methods of comparative analysis, Pearson product moment correlation coefficient and grey relational analysis to explore the situation of grain supply-demand and its balance changes and the influencing factors of grain security, and discussed the influence mechanism of these factors on grain production from three aspects of irrigation rate, multiple cropping index and chemical fertilizer application. In a word, the purpose of this paper was to quantitatively analyze the development trend and spatial distribution characteristics of grain supply-demand, and comprehensively to study the influencing factors of grain security by grey relational analysis. And it provided the reference basis for formulating grain development plans and promoting the steady growth of grain supply.

## Materials and methods

### Data sources

Food, substance consisting essentially of protein, carbohydrate, fat, and other nutrients, is used in the body of an organism to sustain growth and vital processes and to furnish energy [39]. FAO classifies foods as vegetal products and animal products, specifically including cereals, starchy roots, sugar crops, pulses, oilcrops, vegetables, fruits, etc. And cereals and grain belong to the same statistical scope, which include wheat, rice, rye, oats, maize, sorghum, etc. Grain products are processed from various grain crops. For example, rice products include rice, husked rice, flour of rice, milled rice and so on. Wheat products include wheat, meslin flour, breakfast cereals, starch of wheat and so on. Maize products include flour, germ, starch of maize, gluten feed and meal and so on [40, 41]. This paper uses the same statistical range as FAO.

The supply and demand data of grain products are derived from the FAO’s Food Balance Sheet (FBS) documented in the Food and Agriculture Organization Corporate Statistical Database (FAOSTAT). They are the comparatively dependable and conceivably the only database available to pursue and analyze the trends of dietary transition nationally in most countries [42]. Since 2014, the method of FBS has been modified [43, 44]. The new FBS has some methodological advancement in computation compared to the old FBS. The key difference between them is the absence of a balancer variable [45]. In the past, one of the components of the FBS would take on the outstanding unbalanced amounts thus inheriting all the statistical errors. The imputations for the new FBS components are generated by dedicated modules and a balancing mechanism will then proportionally spread the imbalances out among all the components. However, regarding the change of the supply structure and the relationship between supply and demand of grain products in Bangladesh, the methodology of new FBS does not alter this between the old and new series, which they could be combined into an entire time series [46]. Other data on grain crops production, economic development, etc., are from FAOSTAT, the International Bank for Reconstruction and Development (IBRD), the Food Planning and Monitoring Unit (FPMU) database of Ministry of Food Government of Bangladesh and the Statistical Yearbook of Bangladesh. The vector data of administrative divisions are from Natural Earth (http://www.naturalearthdata.com). The digital elevation data are from Shuttle Radar Topography Mission (SRTM) dataset of United States Geological Survey (USGS) (http://eros.usgs.gov/#). Land use data are derived from the global 300 meter resolution climate change initiative land cover (CCI-LC) provided by the European Space Agency (ESA) (http://maps.elie.ucl.ac.be/CCI/viewer/). Flood data come from annual flood report released by the Flood Forecasting and Warming Centre of Bangladesh Water Development Board (http://www.ffwc.gov.bd/) and the Satellite Analysis and Applied Research-UNOSAT (http://floods.unosat.org/).

### Methods

#### Contribution rate of grain

Contribution rate of grain can be divided into three indicators: contribution rate of planting area of grain, contribution rate of production of grain and contribution rate of production increase of grain. It refers to the proportion of planting area, production or production increase of a certain grain crop to the total planting area, total production or production increase of grain. It reflects the importance of various crops to regional grain production in a period [47].

$$v_{i,j}=\frac{p_{i,j}}{p_{i,grain}}*100$$

Where *p*_*i*,*j*_ represents the planting area, production or production increase of the *j* crop in the *i* year, *p*_*i*,*grain*_ represents the planting area, production or production increase of total grain crops in the *i* year, *v*_*i*,*j*_ represents the contribution rate of planting area, production or production increase of the *j* crop in the *i* year, *i* = 1998, 1999, …, 2018, *j* represents the category of grain crops, including rice, wheat, maize and so on.

#### Stability of grain production

Stability of grain production refers to the arithmetic mean value of the proportion of the change value of production of a grain crop in the previous year, which reflects the change degree of this crop production in several years. The positive value represents the increase of grain production. The negative value represents the decrease of grain production. The absolute value represents the range of the increase and decrease of grain production [48].

$$w_{j}=\frac{p_{i+1,j}-p_{i,j}}{p_{i,j}}*100$$

Where *i* represents the statistical year, *i* = 1998, 1999, …, 2018, *j* represents the category of grain crops, including rice, wheat, maize and so on; *p* represents the grain crops production, *w*_*j*_ represents the stability of grain production.

#### Grain self-sufficiency rate

Grain self-sufficiency rate is an index to evaluate the degree of grain self-sufficiency of a country. It represents the ratio between domestic production and demand for different uses of grain products.

$$s_{i,j}=\frac{p_{i,j}}{d_{i,j}}*100$$

Where *i* represents the statistical year, *i* = 1998, 1999, …, 2018, *j* represents the category of grain crops, including rice, wheat, maize and so on, *p* represents the production of domestic grain products, *d* represents the demand of grain products for different uses, *s* represents the grain self-sufficiency rate.

#### Grey relational analysis

Grey relational analysis could solve the complicated interrelationships between multiple factors and variables, i.e., it can analyze uncertain relations between one main factor and all other factors in a given system [38]. The change of grain security situation based on the relationship of grain supply-demand is a typical multi-factor influencing system, and technical, economic and environmental factors may all impede the maintenance and enhancement of grain security [49, 50]. Considering the complexity of the staggered impact of grain supply-demand, this paper quantitatively evaluates the relational degree of grain supply-demand indexes in Bangladesh from the perspective of the components and influencing factors, so as to further analyze the influencing mechanism of grain security. The calculation steps are as follows.

$$\left(X_{1},X_{2},\ldots ,X_{m})=(\begin{array}{cccc} x_{1}(1) & x_{2}(1) & \ldots & x_{m}(1)\\ x_{1}(2) & x_{2}(2) & \ldots & x_{m}(2)\\ \ldots & \ldots & \ldots & \ldots \\ x_{1}(n) & x_{2}(n) & \ldots & x_{m}(n) \end{array}\right)$$

$$y_{i}(k)=\frac{x_{i}(k)}{\frac{1}{n}*\sum_{i=1}^{n}x_{i}(k)}$$

$$Y_{0}(k)=\{y_{0}(1),y_{0}(2),\ldots ,y_{0}(n)\};Y_{i}(k)=\{y_{i}(1),y_{i}(2),\ldots ,y_{i}(n)\}$$

$$\xi_{i}(k)=\frac{{\text{min}}_{i}{\text{min}}_{k}|y_{0}(k)-y_{i}(k)|+\theta *{\text{max}}_{i}{\text{max}}_{k}|y_{0}(k)-y_{i}(k)|}{\left|y_{0}(k)-y_{i}(k)|+\theta *{\text{max}}_{i}{\text{max}}_{k}|y_{0}(k)-y_{i}(k)\right|}$$

$$r(x_{0},x_{i})=\frac{1}{n}*\sum_{k=1}^{n}\xi_{i}(k)$$

Where *x*_*i*_(*k*) is the raw data, *y*_*i*_(*k*) is the normalized data obtained by dimensionless calculation using mean value method, *Y*_0_(*k*) is the reference sequence, *Y*_*i*_(*k*) is the comparison sequence, *ξ*_*i*_(*k*) is grey relational coefficient, *r*(*x*_0_, *x*_*i*_) is grey relational grade, *k* = 1, 2, 3, …, n, *i* = 1, 2, 3, …, m, *θ* is the resolution coefficient, which is 0.5.

#### Pearson product moment correlation coefficient

Pearson product moment correlation coefficient method, also known as Pearson correlation coefficient, is a statistical method that can quantitatively measure the correlation between variables [51]. In this paper, it is mainly used to study the correlation between the yield of main grain crops and agricultural environment indexes, including multiple cropping index, irrigation rate and average chemical fertilizer application per hectare. The basic equation for Pearson correlation coefficient is as follows.

$$r=\frac{\sum_{i=1998}^{i=n}\left(x_{i}-\bar{x})*(y_{i}-\bar{y}\right)}{\sqrt{\sum_{i=1998}^{i=n}{\left(x_{i}-\bar{x}\right)}^{2}}*\sqrt{\sum_{i=1998}^{i=n}{\left(y_{i}-\bar{y}\right)}^{2}}}$$

Where *x*_*i*_ and *y*_*i*_ represent the yield of grain crops and agricultural environment indexes in the *i* year, respectively; $\bar{x}$ and $\bar{y}$ represent the average yield of grain crops and agricultural environment indexes from 1998 to 2018, respectively; *n* = 1999, 2000, …, 2018; *r* represents the Pearson correlation coefficient between the yield of grain crops and agricultural environment indexes.

#### Partial factor productivity and agronomic efficiency of chemical fertilizer

The grain yield depends on the limitation of water and fertilizer conditions. To elucidate the effect of increasing yield of chemical fertilizer in Bangladesh is of great guiding significance for optimizing the level of fertilization, improving the efficiency of nutrient use, exerting the effect of increasing yield of chemical fertilizer and determining the advantageous areas for fertilizer development. Partial factor productivity (PFP) and agronomic efficiency (AE) of applied chemical fertilizer are the international parameter commonly used to characterize the effect of increasing yield [52, 53].

$${\text{PFP}}_{i}=\frac{y_{i}}{f_{i}}$$

$${\text{AE}}_{i}=\frac{y_{i}-y_{1998}}{f_{i}-f_{1998}}$$

Where *y*_i_ is the actual grain yield in the *i* year, *f*_*i*_ is the average fertilizer application per hectare in the *i* year, *i* = 1998, 1999, …, 2018; *y*_1998_ represents the grain yield in 1998, *f*_1998_ represents the average fertilizer application per hectare in 1998.

## Results

### Grain supply

#### Grain crops production

**Fig 2:**
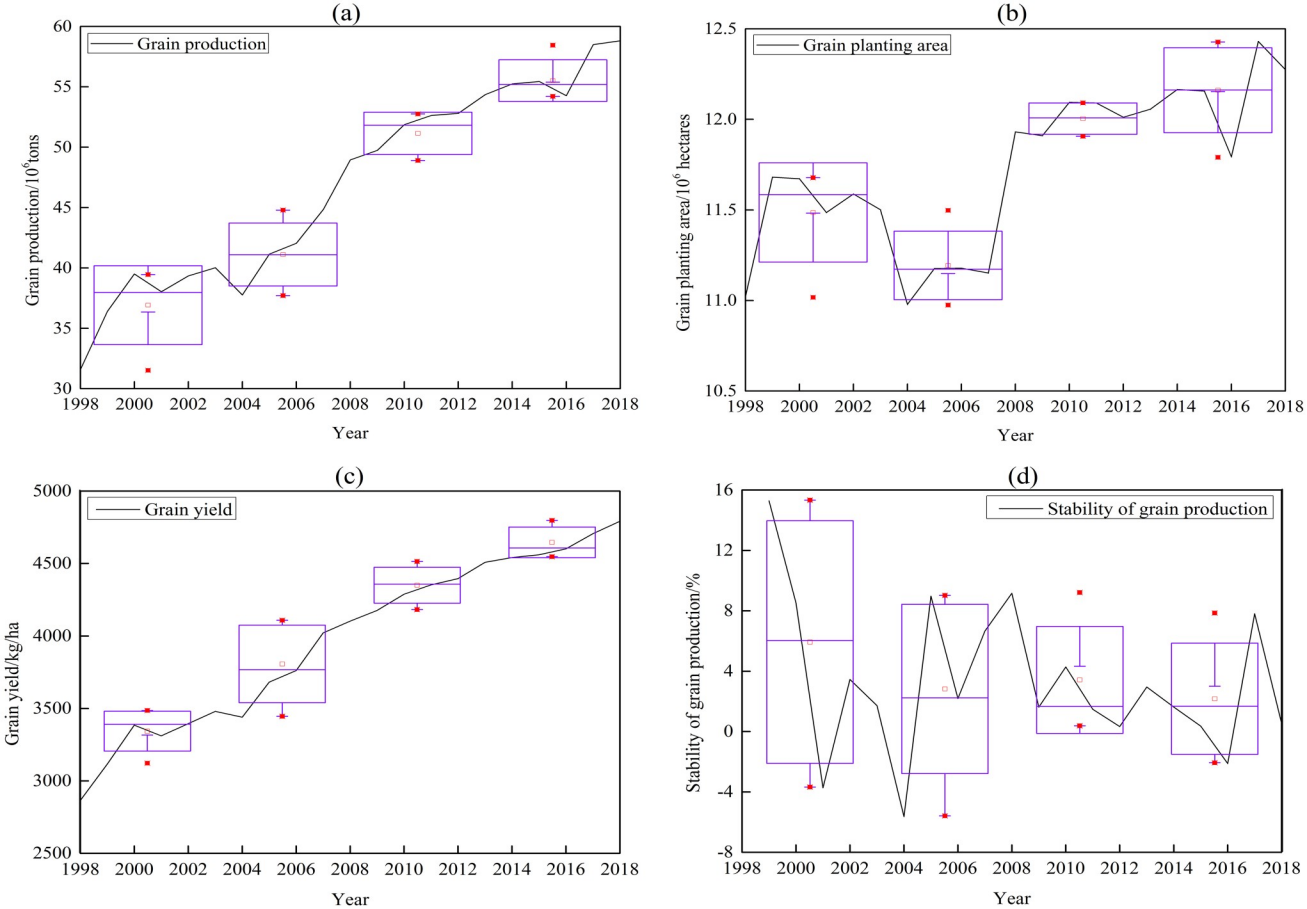
Grain crops production change in Bangladesh from 1998 to 2018. (a) Grain production, (b) Grain planting area, (c) Grain yield, and (d) Stability of grain production. The data of the boxplot are divided into 5 years, which are 1999–2003, 2004–2008, 2009–2013 and 2014–2018 respectively, similarly hereinafter.

Fig 2 showed the grain crops production capacity of Bangladesh was generally good, with the characteristics of high growth rate of production and yield and continuous increase of planting area. From 1998 to 2018, the grain crops production of Bangladesh increased from 31.58 million tons to 58.81 million tons, with an average annual growth rate of 4.11%. However, the average annual growth rate of China’s and India’s grain production was only 1.59% and 1.99%. The growth rate of grain production in Bangladesh was much higher than that of them [41]. The grain yield increased from 2865.3 kg/ha to 4791.2 kg/ha, with an average annual growth rate of 3.2%. Bangladesh’s grain yield in 2018 was only slightly higher than China’s in 1995 and there was still much room for increased grain production in Bangladesh [41]. The grain planting area increased from 11.03 million hectares to 12.27 million hectares, with an average annual growth rate of 0.54%. Natural disasters such as floods and droughts occurred frequently in Bangladesh, especially persistent natural disasters in 1998, 2004–2007 and 2016. The average annual reduction of planting area was about half million hectares during those years, which had a great impact on agricultural production in Bangladesh.

**Fig 3:**
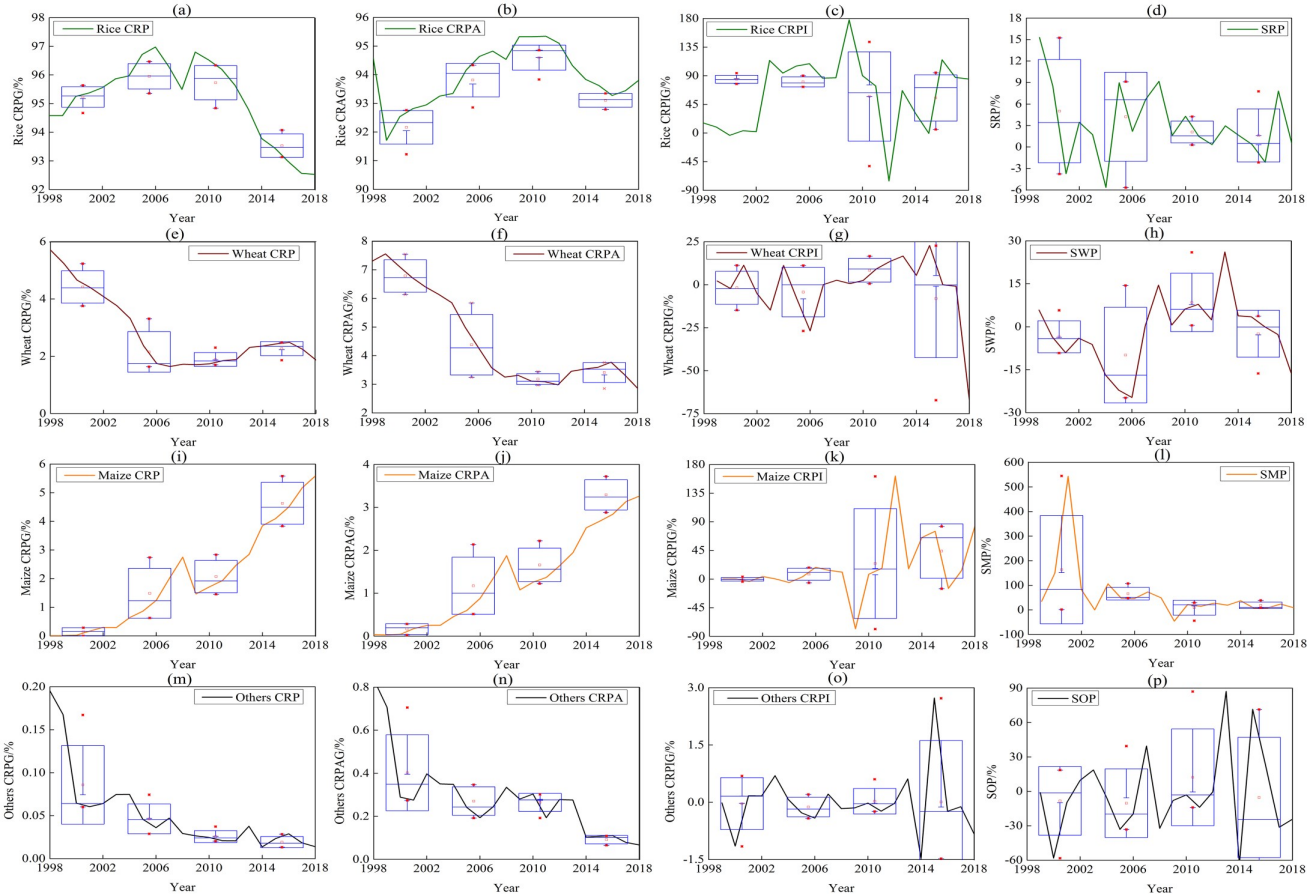
Main grain crops production change in Bangladesh from 1998 to 2018. This figure showed the variation trend of CRP, CRPA, CRPI, SRP, SWP, SMP and SOP in rice (a, b, c, d), wheat (e, f, g, h), maize (i, j, k, l) and others (m, n, o, p) at different periods. (CRP: contribution rate of production, CRPA: contribution rate of planting area, CRPI: contribution rate of production increase, SRP: stability of rice production, SWP: stability of wheat production, SMP: stability of maize production, SOP: stability of others production.

Fig 3 showed that the structure of grain crops was simple and rice, wheat and maize were the main grain crops in Bangladesh. The stability of main crops was lower than that of grain. Rice was the largest grain crop in Bangladesh. It was mainly distributed in the areas of Rangpur division, Mymensingh division, the west of Khulna division and the north of Rajshahi division, including Rangpur, Dinajpur, Naogaon, Mymensingh and Jessore. The annual rice production in each county reached more than 1 million tons. The rice production and planting area have fluctuated in the past 21 years, with an average annual production of about 44.49 million tons and planting area of 11.01 million hectares. The annual contribution rates of production and planting area were about 95.12% and 93.81% respectively. The planting area of wheat decreased continuously, and its contribution rate of production decreased from 5.7% to 1.8%. However, the planting area of maize increased rapidly, and its contribution rate of production increased from 0.01% to 5.59%. With the exception of rice, wheat and maize, the production and planting area of other crops are very small, and their contribution rate has decreased significantly.

#### Supply structure of grain products

**Fig 4:**
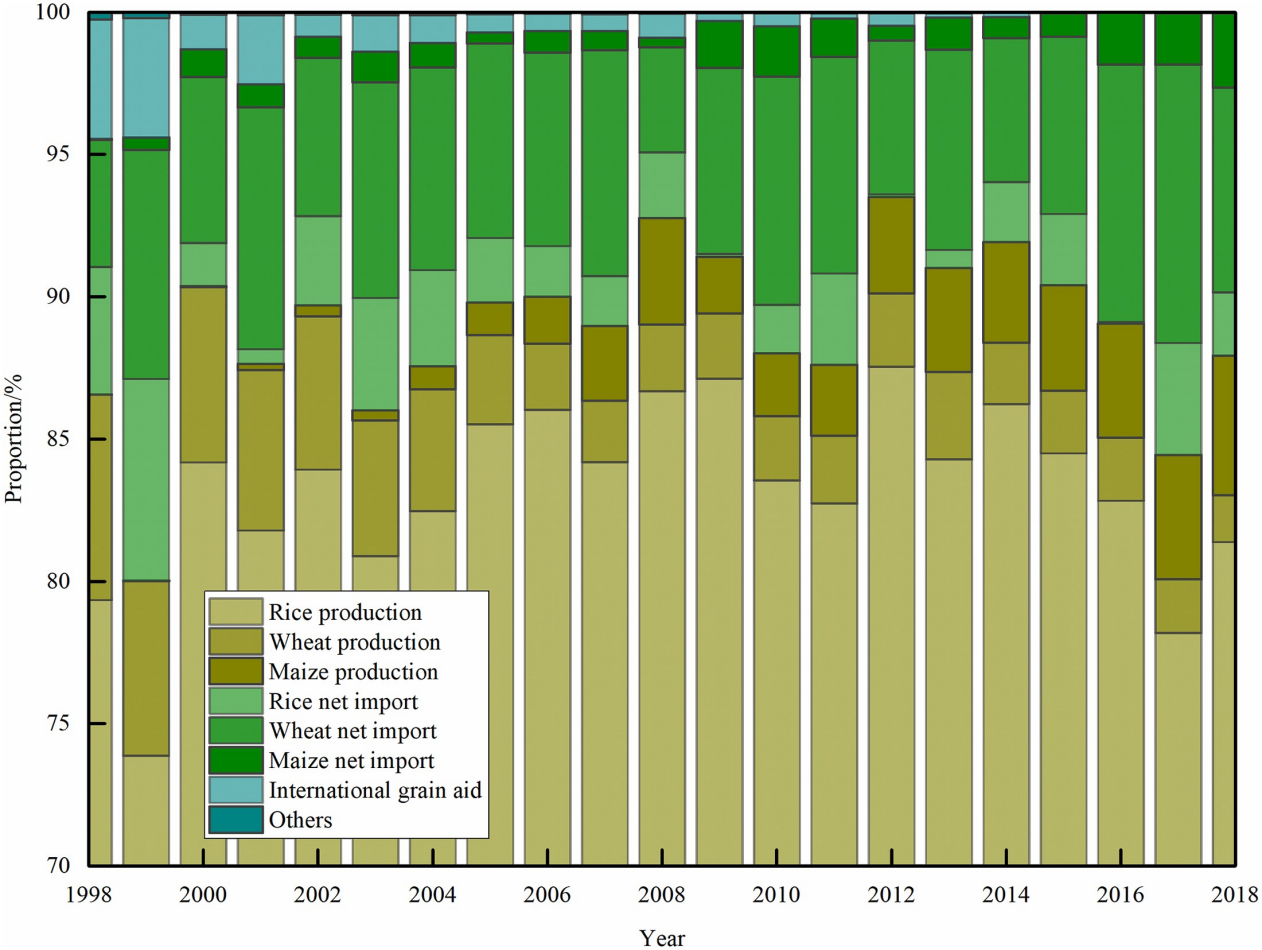
Supply structure of grain products in Bangladesh from 1998 to 2018.

Fig 4 showed that the structure of grain products supply have changed constantly in Bangladesh, with the proportion of rice production, wheat production and international grain aid decreasing, and the proportion of maize production and net grain import increasing. From 1998 to 2018, the supply of grain products increased by 167.69% from 24.98 million tons to 66.87 million tons, with an average annual growth rate of 1.99 million tons. Domestic grain production was the main source of Bangladesh’s grain supply, accounting for an average of 88.86% of total supply. Among them, the proportion of domestic rice production to the total supply was 83.2%. In recent years, with the increase of production, the proportion of domestic maize production gradually exceeded that of wheat. Grain import was the secondary source of grain products supply, accounting for an average of 10.21%. Wheat was the main imported grain, with an annual average of 2.86 million tons. The annual average import of rice and wheat were respectively 0.91 million tons and 0.47 million tons. In addition, international grain aid and other supplies have played a small role in securing Bangladesh’s grain supply.

### Grain demand

#### Grain consumption

With the improvement of the economic level, Bangladesh’s grain consumption structure has been continuously optimized and the diet and nutrition level has been improved. In 1995–2016, the average per capita daily main grain intakes decreased significantly by 110.98 g/day (Table 1), and the proportion of main grain intakes in the total food intakes decreased from 54.5% to 39.67% (Table 2). From the perspective of urban-rural differences, the evolution trend of main grains consumption in urban and rural areas was consistent. However, in rural areas, the decrease was even greater than in urban areas, with the per capita main grain intakes dropping from 81 g/day to 60.61 g/day and the proportion of main grain intakes in food intakes falling from 9.93% to 5.62%. In terms of grain type, rice was the most important grain category, accounting for about 95% of the total grain intakes. The per capita daily rice intakes in rural areas were significantly higher than that in urban areas, with a difference of about 60–90 g/day. There was a small difference between urban and rural areas in per capita daily wheat intakes, both showing a fluctuating downward trend.

**Table 1:** Average per capita daily main grain intakes.

Year	Main grains/(g/day)			Rice/(g/day)			Wheat/(g/day)		
	National	Rural	Urban	National	Rural	Urban	National	Rural	Urban
1995	498	511.4	430.4	464.3	479	390.3	33.7	32.4	40.1
2000	475.7	492.8	402.8	458.5	478.8	372.7	17.2	14	30.1
2005	451.7	467.7	403	439.6	459.7	378.5	12.1	8	24.5
2010	442.01	464.91	377.8	416.01	441.61	344.2	26	23.3	33.6
2016	387.02	403.53	342.92	367.19	386.09	316.7	19.83	17.44	26.22

Data sources: Bangladesh Bureau of Statistics. The main grain in the table included only rice and wheat.

**Table 2:** The proportion and variation of main grain intakes in total food intakes.

Year	Proportion /%			Average annual percent change of main grain intakes/%		
	National	Rural	Urban	National	Rural	Urban
1995	54.50	56.17	46.24	-	-	-
2000	53.26	54.83	46.26	-1.12	-0.91	-1.60
2005	47.66	49.42	42.33	-1.26	-1.27	0.01
2010	44.20	46.25	38.34	-0.54	-0.15	-1.56
2016	39.67	41.42	35.04	-2.49	-1.08	-4.73

Data sources: Bangladesh Bureau of Statistics. The main grain in the table included only rice and wheat.

#### Demand uses of grain products

**Fig 5:**
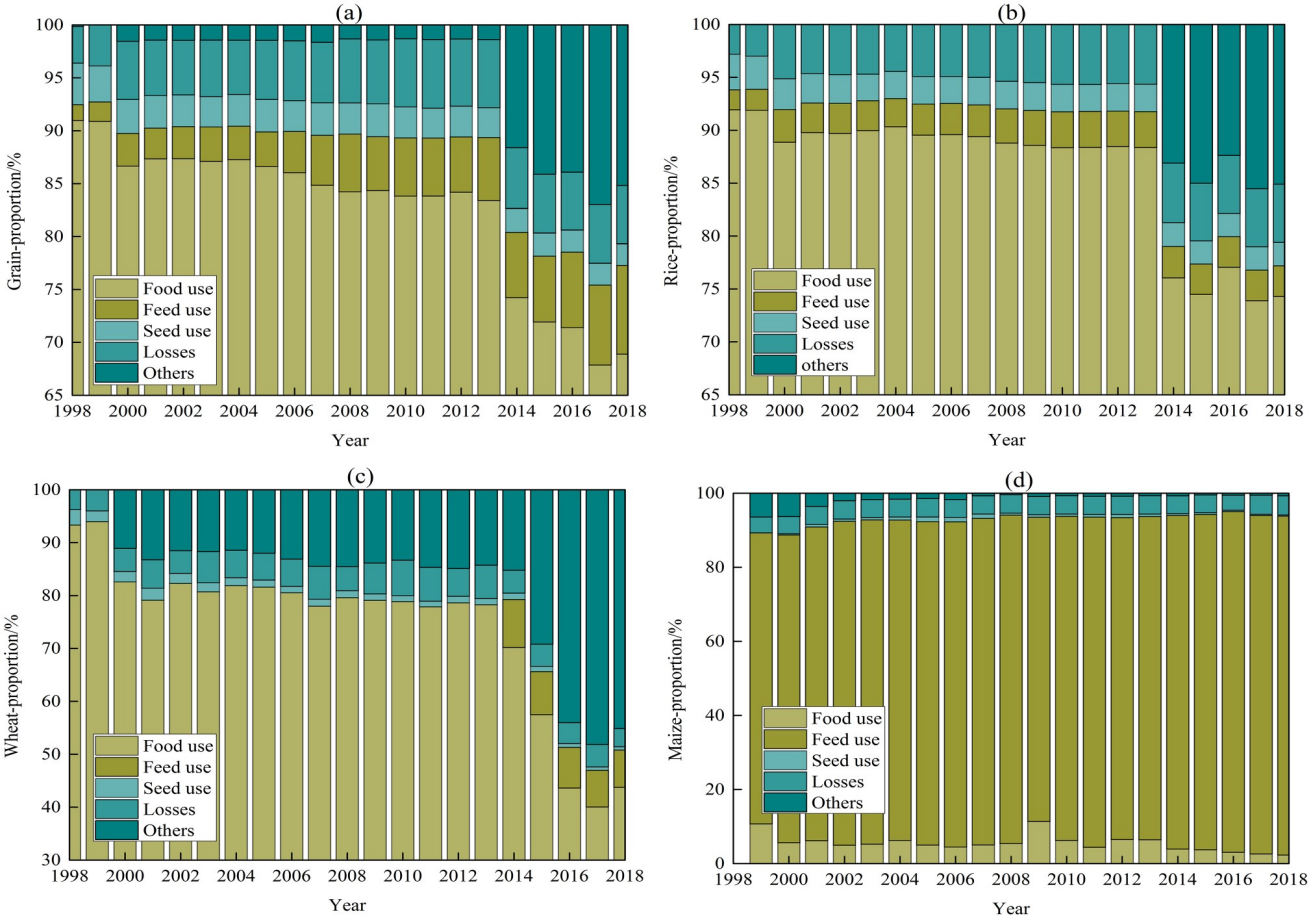
The proportion of demand for different uses of grain products in Bangladesh from 1998 to 2018. (a) Grain products, (b) Rice products, (c) Wheat products and (d) Maize products. The grain demand data for the years 1998–2013 and 2014–2018 are derived from the old and new FBS databases, respectively. The proportion of processing to total demand was too small to be shown in this figure.

The population growth resulted in the apparent increasing trend of grain products demand from 1998 to 2018 in Bangladesh. The population of Bangladesh increased from 123 million to 161 million, an increase of 31 million. The total demand for grain products increased from 24.31 million tons to 68.16 million tons, with an average annual increase of 2.09 million tons, of which rice products from 21.22 million tons to 56.4 million tons, wheat products from 3.02 million tons to 6.88 million tons, and maize products from 11,000 tons to 4.84 million tons. The main grain demand areas of Bangladesh were distributed in the central, northeast and coastal areas, including Dhaka, Mymensingh, Comilla, Sylhet and Chittagong. Even if the demand proportion of grain products for different uses was affected by the old and new FBS databases, it could be concluded that the demand structure of grain products was in a transition period and the uses were increasingly diversified (Fig 5). Food use was the main source of Bangladesh’s grain demand, which the proportion of food use to the total demand went down from 90.96% to 83.4% in 1998–2013 and from 74.21% to 68.88% in 2014–2018. Seed use and losses accounted for a relatively stable, generally 2%-4% and 3%-6.5%. The proportion of feed use and other uses to the total demand showed a continuous growth trend, which the mean of them were respectively 7.09% and 14.35% in 2014–2018. It was in line with the economic development of Bangladesh and the improvement of people’s living standards. In addition, the amount of processed use had increased slightly in recent years, while accounted for an extremely small proportion, about 0.015% in 2014–2018.

### Supply and demand of grain products

**Fig 6:**
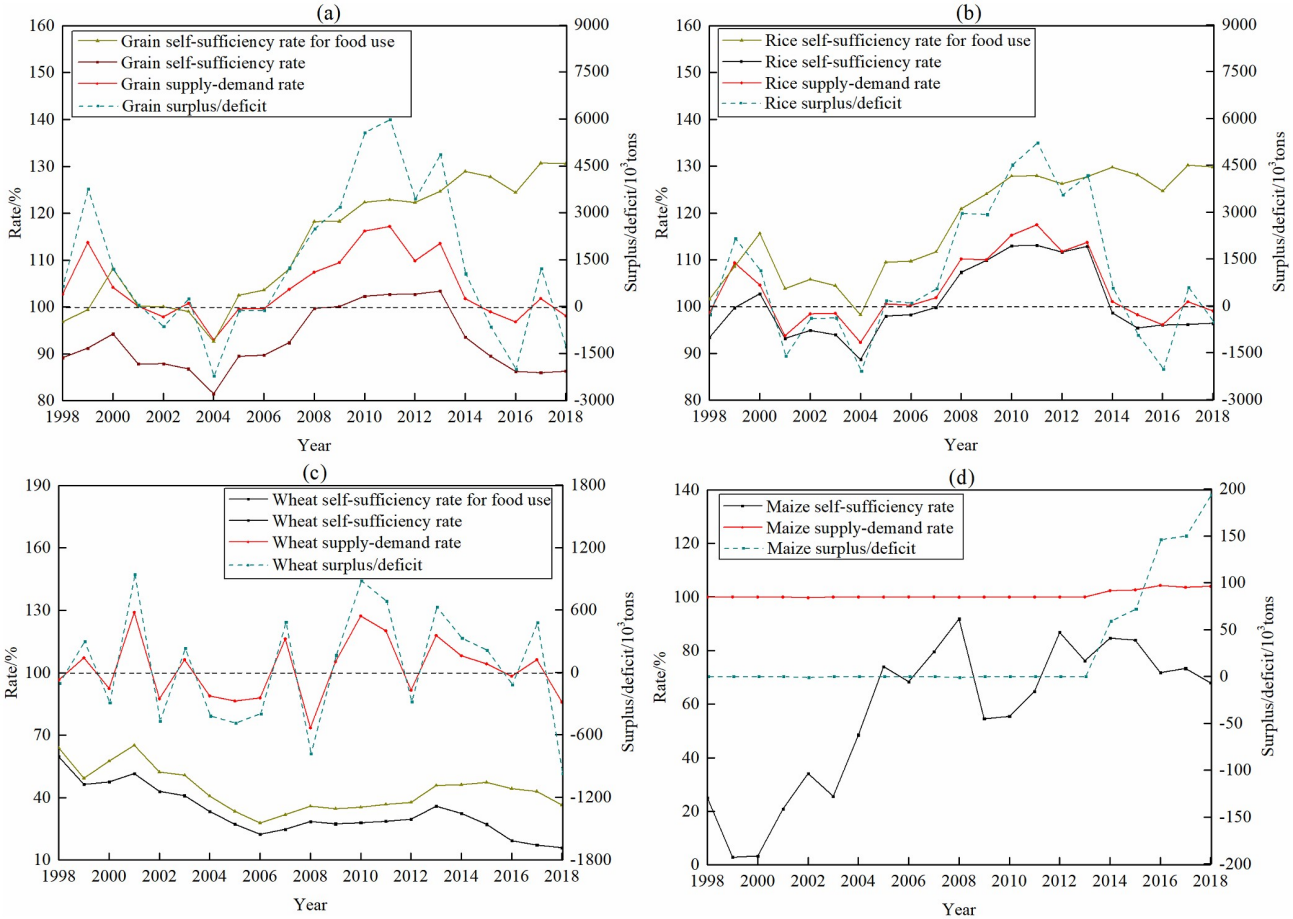
The supply and demand of grain products from 1998 to 2018 in Bangladesh. (a) Grain products, (b)Rice products, (c) Wheat products and (d) Maize products.

The evolution of overall grain supply-demand situation in Bangladesh was divided into three periods: 1998–2006, 2006–2014 and 2014–2018 respectively (Fig 6). From 1998 to 2006, the grain supply-demand situation in Bangladesh was basically balanced, with the average annual grain supply-demand rate of 101.3% and grain surplus of 312,000 tons. The domestic grain production basically met the food use. The average annual grain self-sufficiency rate for food use was 100.26%. From 2006 to 2014, Bangladesh was in grain oversupply, with the average annual grain surplus of 3.33 million tons. The grain supply-demand rate peaked at 117.18% in 2011. From 2014 to 2018, the grain supply and demand situation has worsened, with the average annual grain supply-demand rate of 99.79%. Due to the rapid increase in the grain demand for multiple uses, Bangladesh’s grain dependence on foreign countries has continuously increased. The self-sufficiency rate of grain was reduced to 86.29% in 2018. In addition, there were regional differences in the pattern of grain supply-demand, including surplus grain in the northwest, grain shortage in the middle and Southeast, and balance of supply-demand in other regions. Among them, there were more surplus grain in each county of Rangpur division. The situation of grain shortage in Dhaka and Chittagong divisions was the most serious.

Rice was the most important grain species that dominated the change of grain supply and demand in Bangladesh. The variation trend of rice supply and demand was basically the same as that of grain. Wheat was the highest degree of external dependence of main grain species. From 1998 to 2018, the average wheat self-sufficiency rate was only 32.7%. However, through measures such as grain import and international aid, its overall supply and demand situation showed a fluctuating balance trend. Relative to wheat and rice, the supply and demand situation for maize was the most stable. Except for 2002 and 2008, its balance of supply and demand was maintained in other years.

### Factors affecting grain security

#### The results of grey relational analysis

The relational grade between the factors on the supply side of grain was greater than that on the demand side, with an average value of 0.7602 and 0.6980 respectively in Bangladesh (Table 3). Grain supply had a greater impact on grain security than grain demand. On the supply side, domestic rice production, maize production and imports had the highest relational grade with grain supply-demand balance. These showed that the key to maintaining the grain supply-demand balance in Bangladesh was to ensure the steady growth of domestic grain production, especially rice and maize. The relational grade between agricultural environment indexes and grain production was slightly higher than that between natural resources and agricultural economic indexes and grain production (Table 4). Among them, the multiple cropping index had the greatest impact on grain production. On the demand side, the grain loss and the grain demand for other uses had the greatest impact on the grain surplus/deficit, and their correlation coefficients were 0.6535 and 0.6248 respectively (Table 5). Food and seed use had the least impact. Effectively controlling the degree, quantity and proportion of grain loss and maximizing the reduction of unnecessary grain waste have become an important link to ensure national grain security. The relational grade between socio-economic level index, economic structure and economic development potential and grain demand was 0.7707, 0.8445 and 0.8376. Among them, adjusted net national income per capita had a greater positive effect on the increase in grain demand. And the nutritional structure has been developed in a direction more conducive to health. To sum up, grain production was the top priority to ensure grain security in Bangladesh, among which improving multiple cropping index, irrigation rate of arable land and average fertilizer application per hectare was the most conducive to achieving the balance of grain supply-demand.

**Table 3:** Grey relational analysis results of grain supply-demand balance in Bangladesh.

Relational indexes	Relational grade	Rank	Relational indexes	Relational grade	Rank
Domestic rice production	0.8707	1	Rice net import	0.7349	8
Domestic maize production	0.8025	2	Seed use	0.7086	9
Maize net import	0.7915	3	International grain aid	0.693	10
Losses	0.7633	4	Wheat net import	0.6733	11
Other grain supply	0.7593	5	Food use	0.6535	12
Domestic wheat production	0.756	6	Feed use	0.6248	13
Other uses	0.7399	7			

Note: The maximum difference value between reference sequence and comparison sequence was equal to 4.6195.

**Table 4:** Grey relational analysis results of grain production in Bangladesh.

Relational aspects	Relational indexes	Relational grade	Rank
Agricultural environment	Multiple cropping index	0.7559	1
	Average chemical fertilizer application per hectare	0.6934	4
	Irrigation rate of arable land	0.6712	5
Natural resources	Grain yield	0.7486	2
	Grain planting area	0.643	6
Agricultural economy	Proportion of population in rural areas with access to electricity	0.7222	3
	Proportion of rural population	0.6253	7
	Proportion of population employed in agriculture	0.5618	8
	Agriculture research spending	0.5299	9

Note: The maximum difference value between reference sequence and comparison sequence was equal to 0.52134. Due to the lack of agricultural research spending data for 1998, 1999, 2017 and 2018, the time series of data was 2000–2016 in the grey analysis on grain production.

**Table 5:** Grey relational analysis results of grain demand in Bangladesh.

Relational aspects	Relational factors	Relational grade	Rank
Socio-economic level	Adjusted net national income per capita	0.873	1
	Final consumption expenditure per capita	0.8162	6
	GDP per capita	0.7277	7
	Total population	0.6657	8
Economic structure	Proportion of urban population	0.867	2
	Proportion of non-agriculture industries value added	0.8219	5
Economic growth potential	Annual growth rate of final consumption expenditure	0.8529	3
	Annual growth rate of GDP	0.8226	4

Note: The maximum difference value between reference sequence and comparison sequence was equal to 0.98828.

#### Multiple cropping index affecting grain production

**Fig 7:**
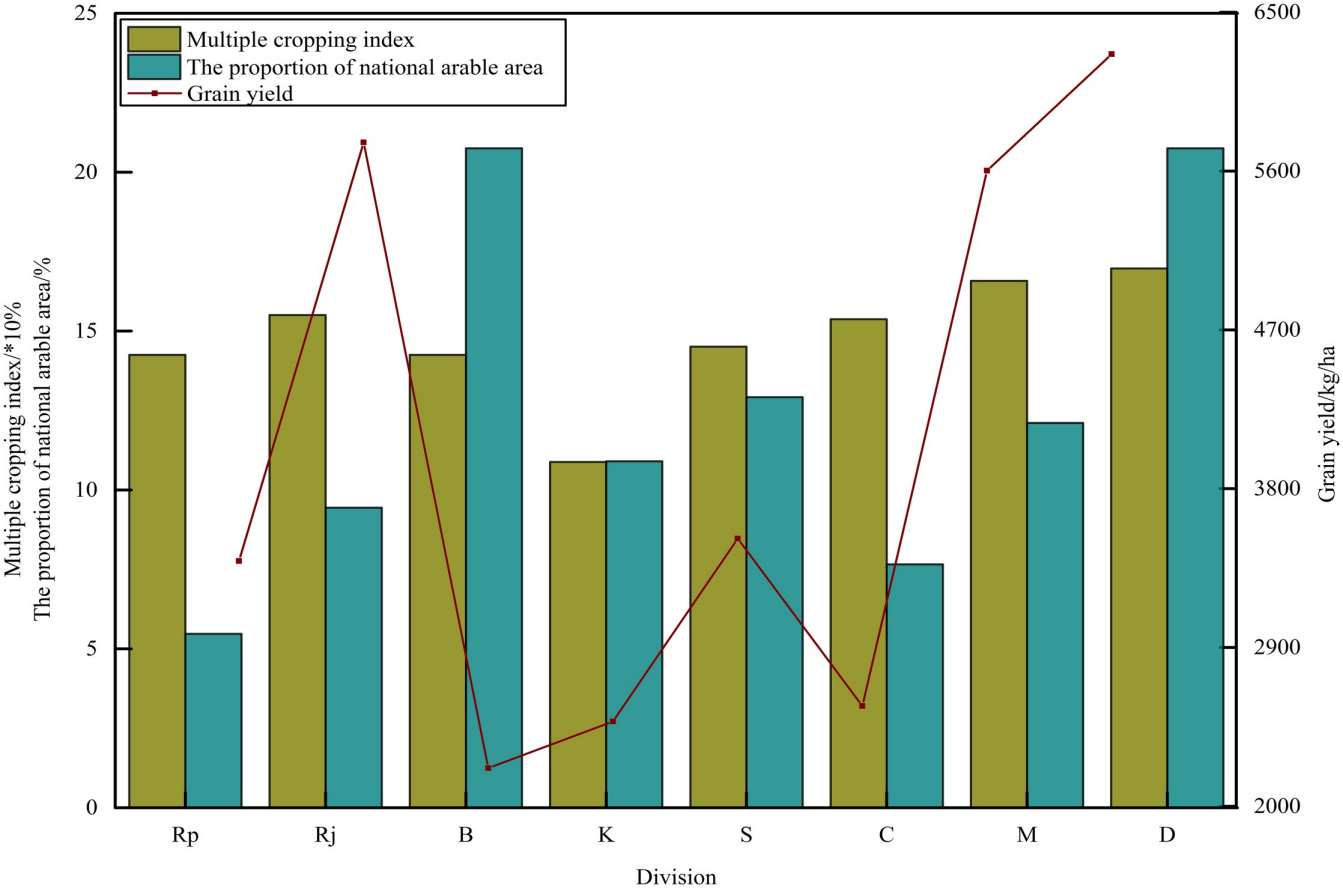
Average of multiple cropping index of eight divisions in Bangladesh from 1998 to 2018. The bar charts of the main coordinate axis in the figure respectively represented the average multiple cropping index of each division and the average proportion of arable area to the national arable area of each division. The line chart of the secondary axis represented the average grain yield of each division. (Rp: Rangpur, Rj: Rajshahi, B: Barisal, K: Khulna, S: Sylhet, C: Chittagong, M: Mymensingh, D: Dhaka).

Multiple cropping was one of the simplest ways to increase regional grain production [54]. According to accumulated temperature conditions and multiple cropping index of cultivated land, the cropping system in Bangladesh could be mainly divided into one cropping cycle per year, three cropping cycles per two years and two cropping cycle per year [55]. From 1998 to 2018, Bangladesh’s grain multiple cropping index increased from 130.36% to 157.93%. There was a close relationship between grain production level and multiple cropping index in different regions of Bangladesh (Fig 7). The multiple cropping index of the main grain producing areas was the highest, with the multiple cropping index of 155% in Rajshahi divisions and 165%-170% in Mymensingh and Dhaka divisions. The grain yield was more than 5500–6500 kg/ha, and the cropping system was three cropping cycles per in two years and two cropping cycle per year. The multiple cropping index was low in other areas with high population density. The multiple cropping index was about 100%-145%. The grain yield was 2000–3500 kg/ha, and the cropping system was one cropping cycle per year and three cropping cycles per in two years. The Pearson correlation coefficient was obtained as follows: grain yield was 0.951 (P<0.001), of which maize was 0.843 (P<0.001), rice was 0.951 (P<0.001) and wheat was 0.76 (P<0.001). It can be seen that the multiple cropping index greatly affected the grain production capacity of the region compared with the arable land area, especially rice production. With the increasingly acute contradiction between human and land in Bangladesh, it was an important measure to increase crop planting area and grain yield by moderately increasing multiple cropping index.

#### Chemical fertilizer application affecting grain production

**Fig 8:**
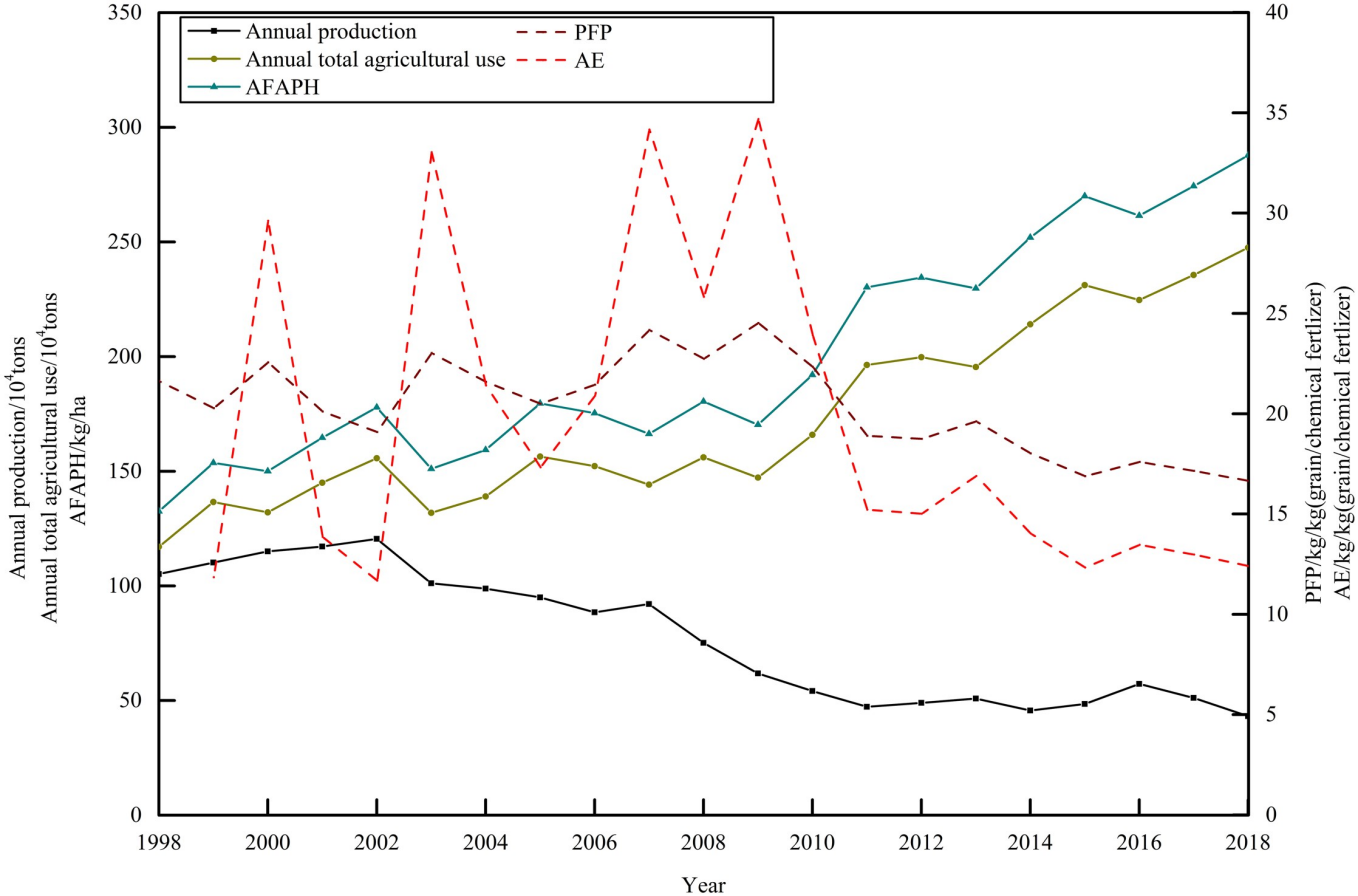
Fertilizer application in Bangladesh from 1998 to 2018. The line charts of the main coordinate axis represented the annual production of chemical fertilizer, the annual total agricultural use of chemical fertilizer and the average fertilizer application rate per hectare (AFAPPH). The line charts of secondary axis represented partial factor productivity (PFP) and agronomic efficiency (AE) of applied chemical fertilizer.

The characteristics of chemical fertilizer application in Bangladesh were low domestic production, large amount of fertilizer application, most of fertilizer being imported, high application rate and weak effect of fertilizer increase (Fig 8). At the beginning of the 20th century, Bangladesh’s self-sufficiency in fertilizer was 80%-90%. In recent years, its fertilizer production capacity has been unable to meet the demand of agricultural production. The fertilizer self-sufficiency rate had fallen to 15%-25%. The annual domestic production capacity was about half million tons, and the imported chemical fertilizer was 1.25–1.45 million tons, accounting for 50%-65% of the total agricultural application. Due to the increase in agricultural fertilizer use in Bangladesh, PFP and AE peaked in 2009, 24.52 kg/kg (grain/chemical fertilizer) and 34.68 kg/kg (grain/chemical fertilizer) respectively. And then they dropped dramatically, to 16.65 kg/kg (grain/chemical fertilizer) and 12.41 kg/kg (grain/chemical fertilizer) respectively in 2018. And the average fertilizer application amount per hectare increased to 287.7 kg/ha in 2018, which was higher than the internationally recognized safe limit of chemical fertilizer application (225 kg/ha). The Pearson correlation coefficient was obtained as follows: grain yield was 0.898 (P<0.001), of which maize was 0.838 (P<0.001), rice was 0.883 (P<0.001) and wheat was 0.87 (P<0.001). It showed that the effect of chemical fertilizer on rice and wheat yield was better than that of maize yield. And there was excessive fertilization in Bangladesh, and the fertilizer application amount had exceeded the optimal application amount in theory. In recent years, the average fertilizer application amount in Bangladesh has been still in a rapid increase in order to maintain the balance of grain supply- demand.

#### Irrigation rate affecting grain production

**Fig 9:**
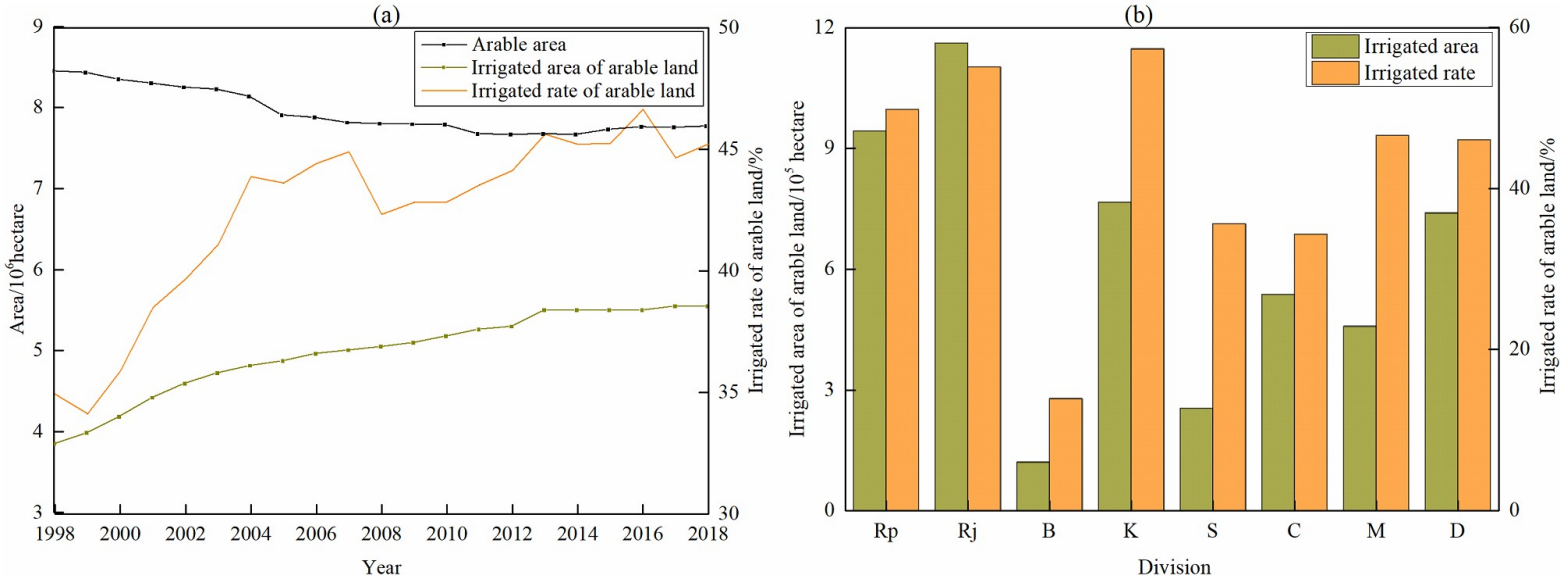
Irrigation change in Bangladesh. (a) Irrigation rate in Bangladesh from 1998 to 2018, (b) Average of irrigation of eight divisions. (Rp: Rangpur, Rj: Rajshahi, B: Barisal, K: Khulna, S: Sylhet, C: Chittagong, M: Mymensingh, D: Dhaka).

The main cultivated land type in Bangladesh was irrigation cropland, which was mainly distributed in the northeast, north and central regions. Rainfed cropland mainly was distributed in the northeast mountainous areas and southern coastal areas. Despite the gradual decrease of arable land area in Bangladesh, the irrigation rate and irrigation area still maintained a steady growth trend (Fig 9). From 1998 to 2018, the overall level of irrigation was relatively high, which the annual average irrigation rate was about twice that of the world average [41]. The arable land area was reduced by 0.68 million hectares from 8.45 million hectares to 7.77 million hectares. The irrigation area of arable land in Bangladesh increased from 3.85 million hectares to 5.55 million hectares. The irrigation rate of arable land increased from 34.93% to 45.22%, with an increase of 10.29%. The Pearson correlation coefficient was obtained as follows: grain yield was 0.836 (P<0.001), of which maize was 0.945 (P<0.001), rice was 0.846 (P<0.001) and wheat was 0.401 (P = 0.072). It can be seen that the continuous improvement of irrigation rate of arable land promoted the increase of grain crop yield, especially maize yield. To ensure and expand irrigation cropland was an important direction of grain production in Bangladesh in the future.

## Discussion

This paper analyzed the development trend and spatial distribution characteristics of grain supply-demand, and comprehensively studied the influencing factors of grain security in Bangladesh. Through exploration and practice, Bangladesh had gradually formed a relatively mature grain security governance system in recent years and had a certain ability to guarantee grain security. Since 1998, the government of Bangladesh has shown a relatively mature level in grain security management. In most years, the relationship of grain supply-demand was in balance or in excess supply. However, Bangladesh still faced many challenges to its grain security, including the gradual reduction of arable land area, the weakening effect of agricultural environmental indicators on increasing grain production, the serious threat of climate change to grain production, the fluctuation of international grain market and the continuous increase of population.

From 1998 to 2018, the area of arable land in Bangladesh decreased from 8.45 million hectares to 7.77 million hectares, and the area of arable land per capita decreased from 0.068 hectares to 0.048 hectares. The massive loss of arable land not only endangered grain security, but also meant increasing grain yield to make up for the loss of arable land. However, Bangladesh’s irrigation rate was about twice the world average, and the average chemical fertilizer application per hectare has exceeded the internationally accepted optimum. Their effect on increasing grain production was waning. Increasing the multiple cropping index was one of the most important measure to maintain the balance of grain supply and demand. However, the multiple cropping index was closely related to climate conditions and economic development, so whether to realize the sustainability of the growth rate of grain planting area and yield remained to be further discussed.

Bangladesh was located in the lower reaches of the Ganges-Brahmaputra-Maghna River Basin, which was the part of the most dynamic hydrological and the biggest active delta system in the world. The main flood disaster was river flood [56]. The inundated areas were centered on the Brahmaputra River, the Maghna River and the Ganges River, including the northeast part of the Rangpur division, the eastern part of Rajshahi and Mymensingh divisions, and most of Dhaka and Sylhet divisions. From 1998 to 2018, an average of about 23% of the land area was affected by flooding each year. In years of severe floods, about 4 million hectares of arable land were affected by floods, accounting for about 50% of the total arable land, such as 1998, 2007 and 2017 [57]. In addition, large areas were affected by rising sea levels in Bangladesh, including Dhaka, Chittagong, Khulna and Barisal divisions. According to the fifth assessment report of the Intergovernmental Panel on Climate Change (IPCC), the rising speed of sea level exceeded the speed of 2.0 mm per year from 1971 to 2010. By the end of the 21st century, the global average sea level will rise by 0.26–0.28 m [58]. According to the forecast of Bangladesh’s grain production capacity in 2018, the cultivated land area with altitude less than 0 m in the coastal divisions was about 47300 hectares, and the average annual grain production would reach about 91600 tons. The cultivated land area of 0-1m above sea level was about 22000 hectares, and the average annual grain production was 4800 tons. The cultivated land area of 1–2 meters above sea level was about 18800 hectares, and the average annual grain production was 40200 tons. Erratic rainfall, droughts and tropical cyclones could also cause problems for agriculture [59, 60]. The extremely poor people in coastal areas were at high risk of survival.

The situation of grain trade in the international market had a great impact on the grain supply of Bangladesh. With the exception of 2009–2013, Bangladesh had to import grain to meet its demand. In 2018, Bangladesh’s grain self-sufficiency rate was 86.29%, and its net grain imports were 8.05 million tons, including 4.81 million tons of wheat, 1.48 million tons of rice and 1.55 million tons of maize. Uncertainties such as global political and public health emergency would have a great impact on the international grain market and then affected Bangladesh’s grain import and export, which would bring certain risks to the balance of grain supply-demand in Bangladesh. The most striking example was the Global Grain Crisis of 2008. Widespread drought had led to a decline in wheat production in Australia and Canada. The expansion of biofuels had led to tight global maize supply. The international grain export prices had risen sharply, coupled with the increase in oil and freight costs over the same period, leading to dramatically rise in the price of basic grain around the world. The grain crisis caused by the change of grain import and export situation swept the world, resulting in global economic recession, international grain prices fluctuated significantly [61, 62]. In the global COVID-19 epidemic outbreak in 2020, the governments of many countries and regions, such as India, Vietnam and Thailand, have introduced policies to intervene in the import and export of grain, and Bangladesh’s grain security was still facing severe challenges.

In addition, the contradiction of grain supply-demand caused by population growth was gradually deepening. It is predicted that the population of Bangladesh will reach 178 million in 2030 and 192 million in 2050. The growth rate will decrease to 0.75% by 2030 and to 0.15% by 2050 [63]. On the whole, although the population growth rate of Bangladesh has slowed down, the population was still growing, with a net annual increase of 968,800 from 2018 to 2050. Based on the standard of 367.19 g/day of rice and 19.83 g/day of wheat per capita in Bangladesh in 2016 [64], Bangladesh will need an additional 129,800 tons of rice and 7,011 tons of wheat per year to meet the grain demand of the new population.

Based on discussions about threats to grain security in Bangladesh, we put forward some suggestions for reference. First, the government of Bangladesh should further strengthen the basic theoretical research and practical application of improving the efficient utilization of agricultural resources through agricultural mechanization, such as precision tillage, precision sowing and precision irrigation. Second, the government of Bangladesh should improve the scientific and cultural quality of the farmers and raise the environmental awareness of the whole people. In combination with national regulation and control policies, the balance between grain yield, environmental protection and product quality should be found. Third, the government of Bangladesh should balance the demand for food use with the need for indirect food and non-food uses. On the basis of satisfying the grain demand for food use of the residents, a certain amount of relatively surplus grain is allowed to be utilized and processed for indirect food and non-food purposes. Finally, the government of Bangladesh should constantly improve the grain circulation system, reduce the loss of grain in the circulation link, and promote the coordinated economic and agricultural development between the exporting and importing regions. The scientific research institutions should continue to strengthen scientific and technological research, reduce grain consumption coefficient or develop non-grain feed and industrial raw materials.

Though based on a long time series, this paper evaluated grain supply-demand changes and grain security influences, and put forward some suggestions, it did have some limitation. This paper mainly used the statistical data of FAOSTAT to calculate the grain supply-demand. The data was of poor timeliness and the spatial analysis was of low precision. Remote sensing image data can be used to study the situation of grain production and get a high-resolution distribution map of grain supply-demand in the following study. And this paper did not deeply analyze the specific factors that affect the change of indicators such as grain yield and planting area. Water resources distribution, climate change and agricultural policy also had a great impact. For example, the change of grain yield can be studied from the factors such as temperature change, precipitation change and extreme weather disasters in the future.

## Conclusions

The grain supply-demand in Bangladesh showed rapid growth, with the annual grain supply increasing to 66.87 million tons and the annual grain demand increasing to 68.16 million tons in 2018. Domestic grain production and food use was the main source of grain supply and demand. Among them, rice, wheat and maize are the main grain crops. Rice was the most important grain species that dominated the change of grain supply and demand. Wheat was the highest degree of external dependence of main grain species. Maize was the most stable for the situation of grain supply-demand. In recent years, Bangladesh’s grain supply-demand situation and self-sufficiency level have been declining. Its dependence of overall grain supply on foreign countries has continuously increased, especially wheat. From the analysis of the influencing factors, domestic rice production, maize production and imports had the highest relational grade with grain supply-demand balance on the supply side and the grain loss and the grain demand for other uses had the greatest impact on the demand side. Improving multiple cropping index, irrigation rate of arable land and average fertilizer application per hectare was the most conducive to increase domestic production for achieving the balance of grain supply-demand. However, faced with the challenges of decreasing arable land area, diminishing impact of agricultural environmental indicators on grain production, climate change, fluctuations in international grain markets and continued population growth, the government of Bangladesh should actively take measures, such as improving the efficient utilization of agricultural resources and grain circulation systems.

## Supporting information

S1 Table Situation of grain crops production in Bangladesh from 1998 to 2018. (PDF)

S2 Table Situation of supply and demand of grain products in Bangladesh from 1998 to 2018. (PDF)

S3 Table Grey relational indexes in grey correlation analysis. (PDF)
