# Supplementary material for: Grain security assessment in Bangladesh based on supply-demand balance analysis
Source: PLoS One. 2021 May 26;16(5):e0252187. doi: 10.1371/journal.pone.0252187 (PMC8153451; doi:10.1371/journal.pone.0252187)
Supplement: S1 Table — (PDF) [file pone.0252187.s001.pdf]

**S1 Table. Situation of grain crops production in Bangladesh from 1998 to 2018.**

| Species | Year | Production | Planting area | Yield | Stability of grain production | Species | Year | Production | Planting area | Yield | Stability of rice production |
|---|---|---|---|---|---|---|---|---|---|---|---|
| | Units | 1000 tons | hectares | kg/ha | % | | Units | 1000 tons | hectares | kg/ha | % |
| Grain | 1998 | 31577.50 | 11020640 | 2865.3 | | Rice | 1998 | 29710.00 | 10119838 | 2935.8 | |
| | 1999 | 36403.00 | 11680974 | 3116.4 | 15.28 | | 1999 | 34430.00 | 10712955 | 3213.9 | 15.89 |
| | 2000 | 39503.00 | 11672247 | 3384.4 | 8.52 | | 2000 | 37627.50 | 10801214 | 3483.6 | 9.29 |
| | 2001 | 38029.34 | 11485595 | 3311 | -3.73 | | 2001 | 36269.00 | 10661000 | 3402 | -3.61 |
| | 2002 | 39341.46 | 11588093 | 3395 | 3.45 | | 2002 | 37593.00 | 10771000 | 3490.2 | 3.65 |
| | 2003 | 40015.28 | 11500907 | 3479.3 | 1.71 | | 2003 | 38361.42 | 10725040 | 3576.8 | 2.04 |
| | 2004 | 37759.05 | 10978274 | 3439.4 | -5.64 | | 2004 | 36235.98 | 10248102 | 3535.9 | -5.54 |
| | 2005 | 41146.76 | 11176338 | 3681.6 | 8.97 | | 2005 | 39795.62 | 10524067 | 3781.4 | 9.82 |
| | 2006 | 42044.69 | 11178034 | 3761.4 | 2.18 | | 2006 | 40773.00 | 10579000 | 3854.1 | 2.46 |
| | 2007 | 44841.30 | 11152085 | 4020.9 | 6.65 | | 2007 | 43181.00 | 10575000 | 4083.3 | 5.91 |
| | 2008 | 48946.80 | 11930855 | 4102.5 | 9.16 | | 2008 | 46742.00 | 11279150 | 4144.1 | 8.25 |
| | 2009 | 49735.85 | 11909888 | 4176 | 1.61 | | 2009 | 48144.00 | 11353532 | 4240.4 | 3.00 |
| | 2010 | 51862.85 | 12093951 | 4288.3 | 4.28 | | 2010 | 50061.20 | 11529000 | 4342.2 | 3.98 |
| | 2011 | 52628.36 | 12090666 | 4352.8 | 1.48 | | 2011 | 50627.00 | 11528000 | 4391.7 | 1.13 |
| | 2012 | 52801.01 | 12011654 | 4395.8 | 0.33 | | 2012 | 50497.00 | 11423000 | 4420.6 | -0.26 |
| | 2013 | 54357.48 | 12056374 | 4508.6 | 2.95 | | 2013 | 51534.00 | 11372000 | 4531.7 | 2.05 |
| | 2014 | 55241.12 | 12165112 | 4540.9 | 1.63 | | 2014 | 51806.59 | 11415642 | 4538.2 | 0.53 |
| | 2015 | 55438.38 | 12156367 | 4560.4 | 0.36 | | 2015 | 51805.46 | 11381221 | 4551.8 | 0.00 |
| | 2016 | 54262.30 | 11793602 | 4601 | -2.12 | | 2016 | 50452.87 | 11000809 | 4586.3 | -2.61 |
| | 2017 | 58495.62 | 12430005 | 4706 | 7.80 | | 2017 | 54148.00 | 11615000 | 4661.9 | 7.32 |
| | 2018 | 58811.62 | 12274921 | 4791.2 | 0.54 | | 2018 | 54416.00 | 11515000 | 4725.7 | 0.49 |

| Species | Year | Production | Planting area | Yield | Stability of wheat production | Species | Year | Production | Planting area | Yield | Stability of maize production |
|---|---|---|---|---|---|---|---|---|---|---|---|
| | **Units** | **1000 tons** | **hectares** | **kg/ha** | **%** | | **Units** | **1000 tons** | **hectares** | **kg/ha** | **%** |
| **Wheat** | **1998** | 1802.82 | 804523 | 2240.8 | | **Maize** | **1998** | 2.97 | 4047 | 733.9 | |
| | **1999** | 1908.00 | 882224 | 2162.7 | 5.83 | | **1999** | 4.00 | 3240 | 1234.6 | 34.68 |
| | **2000** | 1840.00 | 832447 | 2210.4 | -3.56 | | **2000** | 10.00 | 4855 | 2059.7 | 150.00 |
| | **2001** | 1673.00 | 773000 | 2164.3 | -9.08 | | **2001** | 64.34 | 19970 | 3221.6 | 543.35 |
| | **2002** | 1606.00 | 742000 | 2164.4 | -4.00 | | **2002** | 117.26 | 29060 | 4034.9 | 82.26 |
| | **2003** | 1506.71 | 706475 | 2132.7 | -6.18 | | **2003** | 117.26 | 29071 | 4033.4 | 0.00 |
| | **2004** | 1253.38 | 641875 | 1952.7 | -16.81 | | **2004** | 241.46 | 50051 | 4824.3 | 105.93 |
| | **2005** | 975.99 | 558413 | 1747.8 | -22.13 | | **2005** | 356.28 | 66830 | 5331.1 | 47.55 |
| | **2006** | 735.00 | 479000 | 1534.4 | -24.69 | | **2006** | 521.53 | 98404 | 5299.8 | 46.38 |
| | **2007** | 737.00 | 399000 | 1847.1 | 0.27 | | **2007** | 902.15 | 150832 | 5981.2 | 72.98 |
| | **2008** | 844.00 | 388000 | 2175.3 | 14.52 | | **2008** | 1346.47 | 223770 | 6017.2 | 49.25 |
| | **2009** | 849.05 | 394612 | 2151.6 | 0.60 | | **2009** | 729.63 | 128386 | 5683.1 | -45.81 |
| | **2010** | 901.49 | 376256 | 2395.9 | 6.18 | | **2010** | 887.39 | 152009 | 5837.8 | 21.62 |
| | **2011** | 972.09 | 373708 | 2601.2 | 7.83 | | **2011** | 1018.29 | 165542 | 6151.2 | 14.75 |
| | **2012** | 995.36 | 358181 | 2778.9 | 2.39 | | **2012** | 1297.72 | 197108 | 6583.8 | 27.44 |
| | **2013** | 1255.00 | 416416 | 3013.8 | 26.09 | | **2013** | 1548.00 | 234714 | 6595.3 | 19.29 |
| | **2014** | 1303.00 | 429770 | 3031.9 | 3.82 | | **2014** | 2124.00 | 307152 | 6915.1 | 37.21 |
| | **2015** | 1348.00 | 436814 | 3086 | 3.45 | | **2015** | 2272.00 | 325308 | 6984.1 | 6.97 |
| | **2016** | 1348.19 | 444805 | 3031 | 0.01 | | **2016** | 2445.58 | 334974 | 7300.8 | 7.64 |
| | **2017** | 1311.47 | 415339 | 3157.6 | -2.72 | | **2017** | 3025.39 | 389878 | 7759.8 | 23.71 |
| | **2018** | 1099.37 | 351213 | 3130.2 | -16.17 | | **2018** | 3288.10 | 400478 | 8210.4 | 8.68 |
