# Supplementary material for: Grain security assessment in Bangladesh based on supply-demand balance analysis
Source: PLoS One. 2021 May 26;16(5):e0252187. doi: 10.1371/journal.pone.0252187 (PMC8153451; doi:10.1371/journal.pone.0252187)
Supplement: S2 Table — (PDF) [file pone.0252187.s002.pdf]

S2 Table. Situation of supply and demand of grain products in Bangladesh from 1998 to 2018.

S2-1 Table. Situation of supply and demand of grain products in Bangladesh from 1998 to 2018.

Year	Supply	Demand	Surplus/deficit	Production	Net import	Food use	Feed use	Seed use	Losses	Processing use	Other uses (non-food)
**1998**	24978	24315	663	21684	2246	22393	400	805	713	-	4
**1999**	31088	27332	3756	24938	4843	25088	570	805	861	-	8
**2000**	29816	28620	1196	26973	2482	24939	1004	791	1438	-	448
**2001**	29582	29535	47	25952	2915	25911	987	796	1399	-	442
**2002**	29876	30516	-640	26823	2825	26806	1053	789	1431	-	437
**2003**	31635	31386	249	27241	3986	27510	1168	752	1518	-	438
**2004**	29307	31524	-2217	25692	3331	27739	1156	762	1429	-	438
**2005**	31039	31168	-129	27895	2946	27214	1217	760	1534	-	443
**2006**	31614	31732	-118	28467	2952	27479	1486	755	1567	-	445
**2007**	34213	32975	1238	30462	3547	28181	1865	805	1684	-	440
**2008**	35970	33484	2486	33382	2283	28235	2237	805	1770	-	437
**2009**	36863	33675	3188	33704	3059	28498	2064	816	1854	-	443
**2010**	39970	34401	5569	35192	4594	28769	2403	817	1967	-	445
**2011**	40817	34834	5983	35770	4969	29109	2414	810	1984	-	517
**2012**	38475	35030	3445	35987	2318	29428	2311	841	1936	-	514
**2013**	40757	35893	4864	37104	3591	29765	2731	844	2036	-	517
**2014**	60088	59036	1052	55241	4750	42850	4179	1250	3251	13	7493
**2015**	61307	61948	-641	55438	5857	43389	4409	1252	3293	13	9592
**2016**	60910	62906	-1996	54262	6649	43619	5185	1221	3293	9	9579
**2017**	69255	68040	1215	58496	10759	44755	5921	1306	3627	9	12422
**2018**	66870	68162	-1292	58817	8053	45047	6550	1305	3592	14	11654

Note: All data units in the table are 1000 tons and old FBS database did not have statistical data on processing use from 1998 to 2013.

S2-2 Table. Situation of supply and demand of rice products in Bangladesh from 1998 to 2018.

Year	Supply	Demand	Surplus/deficit	Production	Net import	Food use	Feed use	Seed use	Losses	Processing use	Other uses (non-food)
**1998**	20937	21217	-280	19817	1119	19505	399	715	598	-	0
**1999**	25169	23017	2152	22965	2205	21149	459	720	689	-	0
**2000**	25547	24421	1126	25098	449	21702	753	711	1255	-	0
**2001**	24341	25949	-1608	24191	150	23295	726	718	1210	-	0
**2002**	26012	26415	-403	25075	938	23694	752	715	1254	-	0
**2003**	26833	27222	-389	25587	1246	24490	768	684	1280	-	0
**2004**	25156	27240	-2084	24169	987	24604	725	702	1209	-	0
**2005**	27245	27085	160	26544	701	24249	797	706	1328	-	5
**2006**	27756	27672	84	27196	561	24789	816	705	1360	-	2
**2007**	29398	28847	551	28802	597	25786	865	752	1442	-	2
**2008**	32006	29044	2962	31177	830	25787	937	757	1562	-	1
**2009**	32150	29226	2924	32112	38	25884	964	769	1607	-	2
**2010**	34068	29559	4509	33391	676	26113	1003	769	1671	-	3
**2011**	35077	29857	5220	33768	1309	26387	1014	762	1689	-	5
**2012**	33719	30164	3555	33681	38	26681	1011	785	1685	-	2
**2013**	34610	30433	4177	34350	259	26892	1031	788	1718	-	4
**2014**	53059	52488	571	51807	1264	39924	1555	1188	2955	11	6866
**2015**	53326	54261	-935	51805	1533	40429	1555	1188	2955	11	8134
**2016**	50479	52504	-2025	50453	34	40462	1514	1157	2878	8	6493
**2017**	56861	56274	587	54148	2719	41587	1625	1242	3089	7	8731
**2018**	55885	56403	-518	54416	1481	41910	1634	1248	3104	11	8507

Note: All data units in the table are 1000 tons and old FBS database did not have statistical data on processing use from 1998 to 2013.

S2-3 Table. Situation of supply and demand of wheat products in Bangladesh from 1998 to 2018.

Year	Supply	Demand	Surplus/deficit	Production	Net import	Food use	Feed use	Seed use	Losses	Processing use	Other uses (non-food)
**1998**	2920	3024	-104	1803	1117	2822	-	88	114	-	0
**1999**	4409	4114	295	1908	2501	3866	-	83	165	-	0
**2000**	3581	3871	-290	1840	1741	3196	-	77	168	-	430
**2001**	4186	3245	941	1673	2513	2567	-	74	174	-	430
**2002**	3266	3732	-466	1606	1660	3071	-	71	160	-	430
**2003**	3907	3677	230	1507	2400	2967	-	64	216	-	430
**2004**	3339	3758	-419	1253	2086	3076	-	56	196	-	430
**2005**	3095	3582	-487	976	2119	2922	-	48	182	-	430
**2006**	2884	3280	-396	735	2149	2641	-	40	169	-	430
**2007**	3455	2971	484	737	2718	2317	-	39	185	-	430
**2008**	2176	2958	-782	844	1332	2354	-	39	135	-	430
**2009**	3259	3095	164	849	2409	2447	-	38	180	-	430
**2010**	4106	3227	879	901	3205	2544	-	37	216	-	430
**2011**	4081	3396	685	972	3107	2643	-	36	217	-	500
**2012**	3077	3358	-281	995	2081	2639	-	42	177	-	500
**2013**	4125	3498	627	1255	2869	2736	-	41	221	-	500
**2014**	4342	4014	328	1303	3041	2819	364	48	174	1	609
**2015**	5162	4950	212	1348	3816	2847	402	50	207	1	1444
**2016**	6853	6970	-117	1348	5506	3040	536	50	275	2	3069
**2017**	8092	7615	477	1311	6783	3050	527	49	325	2	3664
**2018**	5911	6883	-972	1099	4814	3013	485	41	238	2	3106

Note: All data units in the table are 1000 tons and old FBS database did not have statistical data on processing and feed uses from 1998 to 2013.

S2-4 Table. Situation of supply and demand of maize products in Bangladesh from 1998 to 2018.

Year	Supply	Demand	Surplus/deficit	Production	Net import	Food use	Feed use	Seed use	Losses	Other uses (non-food)
**1998**	11	11	0	3	9	8	0	0	0	3
**1999**	140	140	0	4	136	15	110	1	6	8
**2000**	301	301	0	10	291	17	250	2	14	18
**2001**	308	308	0	64	243	19	260	2	15	12
**2002**	343	344	-1	117	225	17	300	3	17	7
**2003**	458	458	0	117	340	24	400	4	22	8
**2004**	499	499	0	241	256	31	430	6	24	8
**2005**	485	485	0	356	125	24	420	9	24	8
**2006**	766	766	0	522	241	34	670	13	37	12
**2007**	1129	1129	0	902	232	57	1000	8	56	8
**2008**	1466	1467	-1	1346	119	79	1300	9	73	6
**2009**	1339	1339	0	730	609	152	1100	10	66	11
**2010**	1602	1602	0	887	713	100	1400	12	79	11
**2011**	1572	1572	0	1018	553	69	1400	13	77	13
**2012**	1495	1495	0	1298	198	97	1300	13	74	11
**2013**	1948	1948	0	1485	463	125	1700	13	97	13
**2014**	2568	2509	59	2124	444	98	2260	12	121	18
**2015**	2780	2708	72	2272	508	101	2451	13	130	13
**2016**	3554	3408	146	2446	1108	104	3134	13	139	18
**2017**	4275	4125	150	3025	1250	107	3769	14	213	22
**2018**	5035	4841	194	3288	1747	111	4430	15	250	35

Note: All data units in the table are 1000 tons. New and old FBS databases did not have statistical data on processing uses from 1998 to 2018, so it was not shown in the table..
