# Supplementary material for: Grain security assessment in Bangladesh based on supply-demand balance analysis
Source: PLoS One. 2021 May 26;16(5):e0252187. doi: 10.1371/journal.pone.0252187 (PMC8153451; doi:10.1371/journal.pone.0252187)
Supplement: S3 Table — (PDF) [file pone.0252187.s003.pdf]

S3 Table. Grey relational indexes in grey correlation analysis.

S3-1 Table. Grey relational indexes of agricultural environment in grey correlation analysis.

Relational indexes	Multiple cropping index	Average chemical fertilizer application per hectare	Irrigation rate of arable land
**Units**	**%**	**kg/ha**	**%**
**1998**	130.36	132.50	34.93
**1999**	138.47	153.69	34.12
**2000**	139.79	150.03	35.87
**2001**	138.33	164.67	38.49
**2002**	140.41	177.86	39.67
**2003**	139.78	151.09	41.09
**2004**	134.82	159.32	43.88
**2005**	141.28	179.59	43.62
**2006**	141.85	175.38	44.42
**2007**	142.68	166.33	44.90
**2008**	152.90	180.41	42.33
**2009**	152.77	170.29	42.82
**2010**	155.23	192.02	42.83
**2011**	157.47	230.27	43.54
**2012**	156.61	234.46	44.13
**2013**	157.02	229.76	45.62
**2014**	158.63	251.94	45.21
**2015**	157.22	269.99	45.25
**2016**	151.90	261.46	46.64
**2017**	160.24	274.36	44.65
**2018**	157.93	287.74	45.22

S3-2 Table. Grey relational indexes of agricultural economy in grey correlation analysis.

Relational indexes	Proportion of population in rural areas with access to electricity	Proportion of rural population	Proportion of population employed in agriculture	Agriculture research spending
**Units**	**% of rural population**	**annual %**	**% of total employment**	**million PPP (constant 2011 prices)**
**1998**	13.54	79.55	64.93	
**1999**	16.28	79.15	64.94	
**2000**	16.81	78.76	64.81	200.40
**2001**	21.69	78.25	62.39	172.60
**2002**	24.25	77.59	59.90	153.20
**2003**	26.80	76.91	57.19	130.70
**2004**	27.88	76.22	54.30	132.30
**2005**	30.17	75.50	51.17	158.20
**2006**	40.17	74.77	48.08	198.30
**2007**	32.49	74.02	47.85	204.10
**2008**	39.36	73.25	47.67	190.10
**2009**	41.93	72.47	47.53	212.20
**2010**	40.00	71.69	47.31	240.80
**2011**	45.71	70.91	46.55	258.60
**2012**	49.78	70.13	45.76	253.00
**2013**	48.59	69.35	45.01	253.80
**2014**	48.12	68.57	44.27	269.00
**2015**	62.36	67.77	43.46	260.80
**2016**	66.14	66.96	42.66	287.90
**2017**	81.57	66.14	40.60	
**2018**	78.27	65.33	39.39	

S3-3 Table. Grey relational indexes of socio-economic level in grey correlation analysis.

**Relational indexes**	**Adjusted net national income per capita**	**Final consumption expenditure per capita**	**GDP per capita**	**Total population**
**Units**	**constant 2010 US$**	**constant 2011 US$**	**constant 2010 US$**	**1000 persons**
**1998**	470.54	402.58	495.63	122682.8
**1999**	482.06	410.26	508.39	125189.7
**2000**	499.92	422.35	524.95	127657.9
**2001**	514.73	431.58	541.29	130088.7
**2002**	524.82	428.28	551.90	132478.1
**2003**	537.19	437.98	568.14	134791.6
**2004**	570.04	456.69	588.33	136986.4
**2005**	598.92	480.25	617.54	139035.5
**2006**	637.26	507.22	649.93	140921.2
**2007**	675.52	538.28	687.32	142660.4
**2008**	703.22	553.33	720.36	144304.2
**2009**	730.89	559.73	748.30	145924.8
**2010**	769.24	578.95	781.15	147575.4
**2011**	801.76	609.52	822.19	149273.1
**2012**	842.13	627.31	865.75	151005.7
**2013**	879.98	651.89	907.26	152761.4
**2014**	907.58	670.30	951.31	154517.4
**2015**	952.24	701.41	1002.39	156256.3
**2016**	1004.65	714.63	1062.04	157977.2
**2017**	1055.76	759.52	1127.27	159685.4
**2018**	1128.72	834.44	1203.22	161376.7

S3-4 Table. Grey relational indexes of economic structure and economic growth potential in grey correlation analysis.

Relational indexes	Proportion of urban population	Proportion of non-agriculture industries value added	Annual growth rate of final consumption expenditure	Annual growth rate of GDP
**Units**	**annual %**	**annual %**	**annual %**	**annual %**
**1998**	22.82	77.41	1.77	3.01
**1999**	23.20	77.34	3.92	2.57
**2000**	23.59	77.28	4.90	3.26
**2001**	24.10	78.15	4.22	3.11
**2002**	24.76	79.42	1.53	1.96
**2003**	25.43	80.19	4.33	2.94
**2004**	26.11	80.73	6.21	3.55
**2005**	26.81	81.43	6.94	4.97
**2006**	27.52	81.97	7.12	5.24
**2007**	28.24	82.19	7.13	5.75
**2008**	28.97	82.40	3.93	4.81
**2009**	29.71	82.90	2.54	3.88
**2010**	30.46	83.00	4.76	4.39
**2011**	31.23	83.19	6.51	5.25
**2012**	31.99	83.82	4.04	5.30
**2013**	32.76	84.51	5.17	4.79
**2014**	33.54	84.65	4.28	4.86
**2015**	34.31	85.22	6.03	5.37
**2016**	35.08	85.95	3.40	5.95
**2017**	35.86	86.59	7.45	6.14
**2018**	36.63	86.93	11.36	6.74
